# Oxidative stress downstream of mTORC1 but not AKT causes a proliferative defect in cancer cells resistant to PI3K inhibition

**DOI:** 10.1038/onc.2016.435

**Published:** 2016-12-19

**Authors:** M Dermit, P Casado, V Rajeeve, E H Wilkes, D E Foxler, H Campbell, S Critchlow, T V Sharp, J G Gribben, R Unwin, P R Cutillas

**Affiliations:** 1 Cell Signalling & Proteomics, Centre for Haemato-Oncology, Barts Cancer Institute, Queen Mary University of London, London, UK; 2 Centre for Molecular Oncology, Barts Cancer Institute, Queen Mary University of London, London, UK; 3 AstraZeneca, Oncology iMED, Cheshire, UK; 4 Cancer Immunology Group, Centre for Haemato-Oncology, Barts Cancer Institute, Queen Mary University of London, London, UK; 5 UCL Centre for Nephrology, Royal Free Campus and Hospital, University College London, London, UK

## Abstract

Compounds targeting phosphatidylinositol-3-kinase/mammalian target of rapamycin (PI3K/mTOR) signaling are being investigated in multiple clinical settings, but drug resistance may reduce their benefit. Compound rechallenge after drug holidays can overcome such resistance, yet little is known about the impact of drug holidays on cell biochemistry. We found that PI3K inhibitor (PI3Ki)-resistant cells cultured in the absence of PI3Ki developed a proliferative defect, increased oxygen consumption and accumulated reactive oxygen species (ROS), leading to lactate production through hypoxia-inducible factor-1α. This metabolic imbalance was reversed by mammalian target of rapamycin complex 1 (mTORC1) inhibitors. Interestingly, neither AKT nor c-MYC was involved in mediating the metabolic phenotype, despite the latter contributing to resistant cells' proliferation. These data suggest that an AKT-independent PI3K/mTORC1 axis operates in these cells. The excessive ROS hampered cell division, and the metabolic phenotype made resistant cells more sensitive to hydrogen peroxide and nutrient starvation. Thus, the proliferative defect of PI3Ki-resistant cells during drug holidays is caused by defective metabolic adaptation to chronic PI3K/mTOR pathway inhibition. This metabolic imbalance may open the therapeutic window for challenge with metabolic drugs during drug holidays.

## Introduction

Phosphatidylinositol-3-kinase/AKT/mammalian target of rapamycin (PI3K/AKT/mTOR) signaling has key roles in the regulation of cell growth, survival, motility and bioenergetic metabolism, and it is one of the most frequently mutated pathways in cancer.^1^ Consequently, small-molecule inhibitors targeting the PI3K pathway are being developed at a rapid pace, and both preclinical and early clinical studies are beginning to suggest strategies for their effective therapeutic use.^2^ Experience with other successful targeted agents, however, suggests that resistance is likely to reduce the durability of any clinical benefit.^3, 4^

The drug holiday strategy (drug removal followed by rechallenge) has been successfully used to overcome resistance in melanoma, chronic myeloid leukemia and lung cancer cells treated with the kinase inhibitors vemurafenib, imatinib and erlotinib, respectively.^5, 6, 7^ In a heterogeneous tumor environment, resistant cells develop a proliferative disadvantage during drug removal, resulting in their replacement by sensitive cells. The proliferative disadvantage suffered by resistant cells in the absence of drug is considered as a key event for the success of this strategy.^6^ The molecular mechanisms that give rise to this deficit in proliferation are poorly understood, and a better knowledge could be used to develop strategies to improve the response of patients treated with signaling inhibitors.

The overactivation of the c-Myc oncogene has been identified as a mechanism of acquired resistance to PI3K inhibition in several preclinical studies.^8, 9, 10^ Resistance to inhibitors of the PI3K/AKT/mTOR axis may also arise by the activation of parallel pathways, such as RAF/MEK/ERK^11^ and EGFR/PKC (epidermal growth factor receptor/protein kinase C) signaling axes.^12^

Here, we aimed to understand the adaptations that occur in cells with acquired resistance to PI3K/mTOR inhibitors and the impact of drug holidays on cell biochemistry. We found that resistant cells adapted their metabolic homeostasis to compensate for chronic PI3K pathway inhibition and underwent profound metabolic changes after drug deprivation (that is, in drug holidays conditions). Interestingly, these alterations included an increase of glycolytic activity that in other systems is known to promote cell proliferation.^13^ The accumulation of reactive oxygen species (ROS), however, not only prevented resistant cells from recovering the division rate of parental cells but was also detrimental to their proliferation. We found that ROS were produced in a mammalian target of rapamycin complex 1 (mTORC1)-dependent, but AKT-independent, manner and mediated glycolytic activity via hypoxia-inducible factor (HIF), but not c-MYC. Our results suggest that a metabolic imbalance is not only a hallmark of cancer, but it also causes resistant cancer cells on drug holidays to acquire a proliferative defect that could be enhanced with additional oxidative challenge.

## Results

### Cells with chronic inhibition of PI3K develop a proliferative defect and a hypermetabolic phenotype during drug holidays

**Figure 1:**
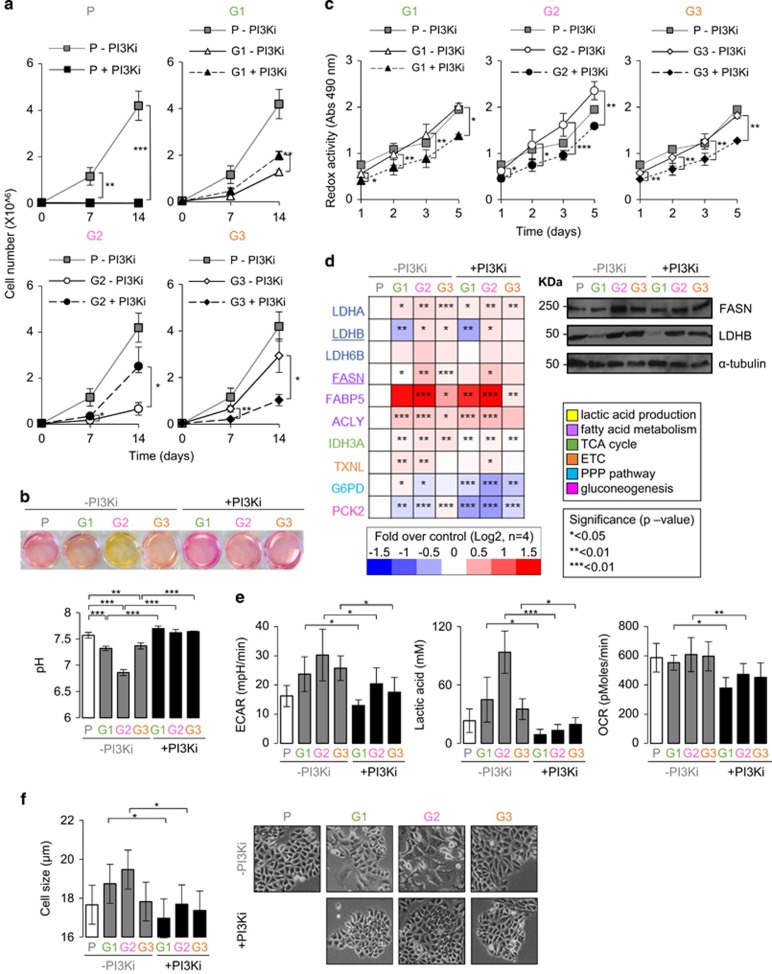
PI3Ki-resistant cells in drug holidays develop a proliferative defect and a hypermetabolic phenotype. (**a**) Cell proliferation measured after 7 and 14 days of growing in the absence or presence of 1 μM GDC-0941 (PI3Ki). G1, G2, G3, PI3Ki-resistant MCF7 cells; P, parental MCF7 cells. (**b**) Images and pH values of media collected from cells at day 5. (**c**) Redox activity as determined by the MTS assay. (**d**) Expression of proteins involved in several metabolic processes. Western blot images of fatty acid synthetase (FASN) and lactate dehydrogenase B (LDHB; right image). (**e**) ECAR and OCR and media lactic acid measurements. (**f**) Representative images and diameter size of cells. Data are represented as mean±s.d. (*n*⩾3, independent biological replicates). *P*-values were calculated using an unpaired, two-tailed Student's *t*-test comparing as indicated; *P<0.05; **P<0.01; ***P<0.001.

To investigate the biochemical adaptations that occur in cells with acquired resistance to PI3K inhibition, we used three independent cell lines (named G1, G2 and G3) derived from chronic treatment of the MCF7 cell line with the PI3K class IA-specific inhibitor GDC-0941 (PI3Ki, Figure 1a and Supplementary Figure S1a).^14^ Resistant cells were able to proliferate—although at slower rate than parental cells—in the presence of 1 μM of compound, whereas parental cells could not (Figure 1a and Supplementary Figure S1a). Of note, none of the resistant cells recovered the proliferation rate of the parental cells upon drug withdrawal (Figure 1a). Interestingly, G1 and G2 grew even slower in the absence rather than in the presence of the drug (Figure 1a). These data suggest that PI3Ki-resistant cells have developed a proliferative defect that is manifested during drug holidays, with G1 and G2 even showing a potential addiction to the PI3Ki.

Drug withdrawal caused a substantial acidification of the media (that was greater in G2), suggesting a metabolic alteration (Figure 1b). This difference in metabolic rate was associated with an increased redox activity after compound withdrawal (Figure 1c), and was a reversible process, as drug readministration restored the initial phenotype (Supplementary Figure S1b).

To determine the enzymes involved in the metabolic phenotype, we measured the transcriptional expression of metabolic genes. This analysis showed a different expression pattern of metabolic enzymes between parental and resistant cells (Supplementary Figure S1c). Mass spectrometry-based proteomics analysis showed that resistant cells increased the expression of lactate dehydrogenase A, fatty acid synthetase, isocitrate dehydrogenase 3A and thiorredoxin-like protein that are involved in production of lactic acid, fatty acid metabolism, tricarboxylic acid cycle and electron transport chain, respectively. On drug removal, resistant cells expressed proteins involved in the pentose phosphate pathway and gluconeogenesis, including glucose-6-phosphate dehydrogenase and phosphoenolpyruvate carboxykinase 2, respectively (Figure 1d). Immunoblotting analysis confirmed the expression changes of fatty acid synthetase and lactate dehydrogenase B (Figure 1d).

To characterize the metabolic alterations in more detail, we measured mitochondrial (oxygen consumption rate (OCR)) and glycolytic (extracellular acidification rate (ECAR)) activities as well as lactic acid levels in the media (Figure 1e). We found that drug removal produced an increase in both ECAR and lactic acid in all resistant cells (Figure 1e). OCR was also significantly increased upon drug removal in G1 and G2; however, in G3 this increase did not reach statistical significance (Figure 1e). Taken together, these results indicate that placing PI3Ki-resistant cells in drug holiday conditions induces a hypermetabolic phenotype characterized by an increase in both glycolysis and mitochondrial respiration.

We observed that the development of the metabolic phenotype was associated with an increase in cell size (Figure 1f), and with a slow cell cycle progression from G_0_/G_1_ to S phase (Supplementary Figure S1d) without affecting apoptosis (Supplementary Figure S1e). These data indicate that the drug holiday-induced metabolic changes contributed to the production of cell biomass, but instead of being used to proliferate, PI3Ki-resistant cells diverted such biomass to increase size.

### PI3Ki-resistant cells in drug holidays reactivate PI3K/mTORC1 signaling that mediates the hypermetabolic phenotype independently of AKT

**Figure 2:**
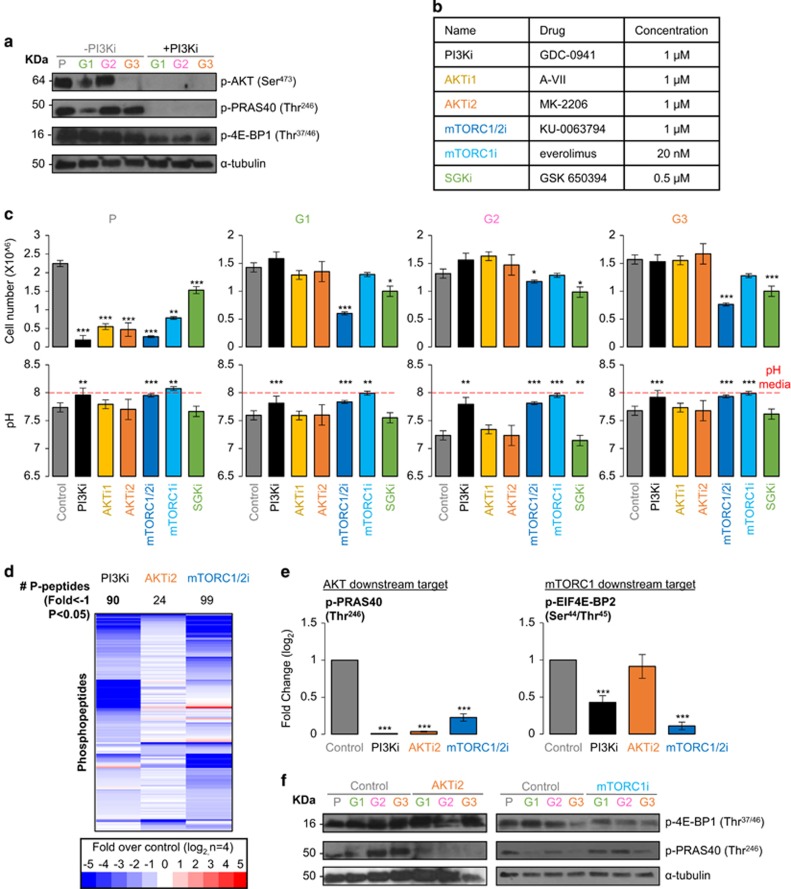
Drug withdrawal from PI3Ki-resistant cells causes an AKT-independent reactivation of mTORC1 that regulates a hypermetabolic phenotype. (**a**) Phosphorylation of PI3K/AKT/mTORC1 axis activity markers in cells. (**b**) Inhibitors used in experiments shown in (**c**). (**c**) Cell numbers and media pH values in cells as a function of treatment with the named inhibitors for 5 days. Data are represented as mean±s.d. (*n*=3, three independent biological replicates). (**d**) Phosphopeptides significantly modulated in G2 cells treated for 1 h with the named inhibitors. (**e**) Detailed quantification of PRAS40 and EIF4E-BP2 phosphopeptides. (**f**) Immunoblot images of the named phosphorylation sites as a function of treatment. *P-*values calculated using an unpaired, two-tailed Student's *t*-test between treatment and control; *P<0.05; **P<0.01; ***P<0.001.

Markers of mTORC2, AKT and mTORC1 activities (namely phosphorylations at AKT Ser^473^, PRAS40 Thr^246^ and 4E-BP1 Thr^37/46^, respectively) remained inhibited in resistant cells growing in the presence of PI3Ki (Figure 2a and Supplementary Figure S2a). On drug holidays, phosphorylation on these markers dramatically increased relative to the same cell line growing exposed to the PI3Ki, although the extent of such reactivation was different across G1, G2 and G3 cell lines (Figure 2a and Supplementary Figure S2a).

To determine the components involved in the development of the metabolic phenotype, we measured cell viability and media acidification as a function of treatment with inhibitors of the PI3K cascade^15, 16, 17, 18, 19^ (Figure 2b). PI3Ki-resistant cells were crossresistant to AKT and mTORC1/2 inhibition (Figure 2c, top panels), indicating that these cells had developed the ability to survive in the absence of PI3K/AKT/mTOR pathway activity.

The mTORC1/2 and mTORC1 inhibitors prevented media acidification in all resistant cell lines (Figure 2c, bottom panels). For example, G2 acidified the media to pH 7.2 (normal media pH is 8.0); the pH of cells treated with PI3Ki, mTORC1/2i or mTORC1i remained at pH of 7.8, 7.8 and 8.0, respectively (Figure 2c, bottom panel). Surprisingly, we found that G2 cells treated with two different AKT inhibitors acidified the media to the same extent as untreated cells (pH 7.3 and 7.2). Similar results for pH values were observed for the G1 and G3 cell lines (Figure 2c, bottom panels). Inhibition of SGK (serum- and glucocorticoid-inducible kinase) with GSK-650394 ^(ref.^^20^) did not prevent the metabolic phenotype (Figure 2c, bottom panels). Thus, SGK, a signaling mediator that can occasionally substitute AKT,^21^ did not replace AKT signaling in our model of drug resistance.

To investigate the relevance and extent of AKT-independent PI3K/mTOR signaling in our model, we compared the effects of PI3Ki, mTORC1/2i and AKTi2 on the phosphoproteome of G2 cells (Figure 2d and Supplementary Figures S2b and c). PI3Ki and mTORC1/2i inhibited the phosphorylation of 90 and 99 sites, respectively, whereas AKTi2 only inhibited the phosphorylation of 24 sites (at *P*<0.05, log_2_ fold <−1, *n*=4, Figure 2d). Of note, PI3K and mTORC1/2 inhibitors, but not the AKTi2, reduced the phosphorylation of the EIF4E-BP2 (Ser^44^/Thr^45^), a downstream mTORC1 target (Figure 2e). In contrast, mTORC1/2, AKT and PI3K inhibitors blocked the phosphorylation of the AKT substrate PRAS40 (Thr^246^), as determined by liquid chromatography–mass spectrometry (Figure 2e). Western blot analysis also showed that PRAS40 (Thr^246^) phosphorylation was inhibited by AKTi. The mTORC1 inhibition with everolimus reduced the phosphorylation of 4E-BP1 (Thr^37/46^), whereas AKTi did not reduce the phosphorylation at this site (Figure 2f). Together, these data demonstrate that an AKT-independent PI3K/mTORC1 signaling axis is functional in our cell model of drug resistance and that this pathway is involved in the mediation of the metabolic phenotype.

### c-MYC, CAMKII and MEK activities have a role in the viability of PI3Ki-resistant cells but are not responsible for the hypermetabolic phenotype

**Figure 3:**
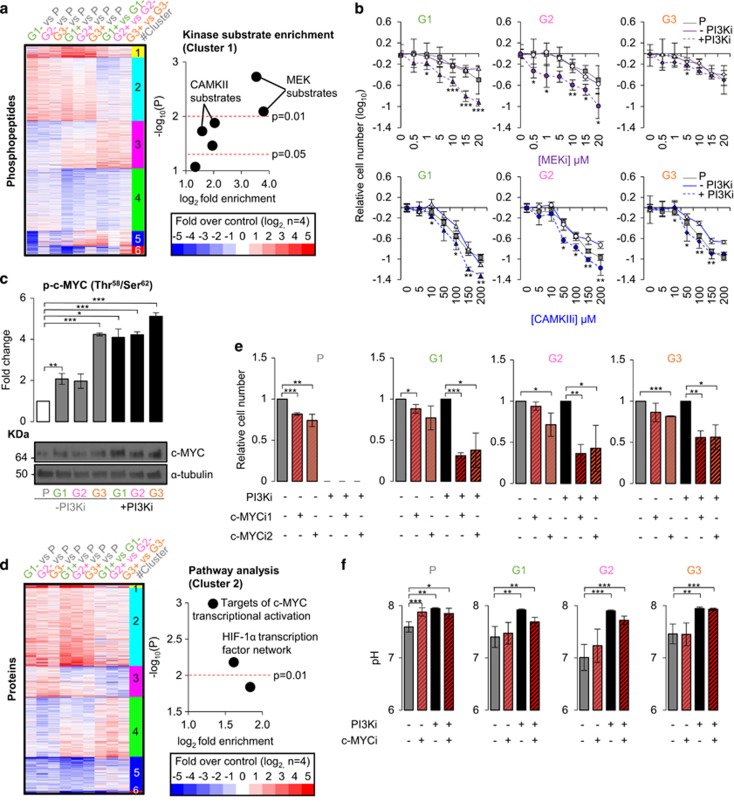
c-MYC, CAMKII and MEK contribute to the viability of PI3Ki-resistant cells but these proteins do not control the hypermetabolic phenotype. (**a**) *K*-means clustering of phosphopeptides significantly modulated by chronic PI3Ki treatment. Right panel shows kinase substrate enrichment analysis (KSEA) of phosphopeptides increased in resistant cells (cluster 1). (**b**) Relative cell numbers as a function of trametinib or KN-93 treatment in the presence or in the absence of PI3Ki (*n=*3). (**c**) Phosphorylation of p-c-MYC (Thr^58^/Ser^62^) and protein levels of c-MYC. Phosphorylation data are represented as mean±s.d. (**d**) *K*-means clustering of fold changes (log_2_) for proteins significantly modulated by chronic PI3Ki treatment. Right panel shows pathway analysis of entries enriched in PI3Ki-resistant cells relative to parental (Cluster 2). (**e**) Relative cell numbers as a function of treatment with 1 μM PI3Ki with either 25 μM 10058-F4 (c-MYCi1) or 20 μM 10074-G5 (c-MYCi2) for 5 days. (**f**) Media pH from cells cultured for 5 days with DMSO control, PI3Ki c-MYCi or the combination. Unless indicated, data are represented as mean±s.d. (*n*=3). *P*-values calculated using an unpaired, two-tailed Student's *t*-test comparing drug-resistant cells growing in the presence or absence of PI3Ki for (**b**), and as indicated for (**c**), (**e**) and (**f**); *P<0.05; **P<0.01; ***P<0.001.

To better understand signaling changes in resistant cells, we analyzed the phosphoproteomes of parental and resistant cells in the presence or absence of PI3Ki (Supplementary Figure S3a). Of the 5674 phosphopeptides quantified in these experiments, 2166 were significantly altered (at *P*<0.05 and log_2_ fold <−1 or >1) in at least one condition. Unsupervised clustering (using a *k*-means algorithm) grouped phosphopeptides that significantly increased (clusters 1–2) or decreased (4 and 6) in resistant compared with parental cells (Figure 3a and Supplementary Dataset S2). Substates for MEK and CAMKII were enriched in cluster 1, suggesting that these kinases were more active in resistant relative to parental cells (Figure 3a and Supplementary Figure S3b). To determine whether MEK and/or CAMKII could mediate resistance to PI3K inhibition, we measured proliferation of resistant cells in the presence of trametinib (a MEK inhibitor)^22^ or KN-93 (a CAMKII inhibitor).^23^ As predicted, PI3Ki potentiated the effects of trametinib and KN-93 in PI3Ki-resistant cells (Figure 3b). However, treatment with trametinib or KN-93 did not avoid the appearance of the metabolic phenotype (Supplementary Figure S3c).

Of interest, Figure 3c shows that resistant cells increased the phosphorylation of c-MYC at sites (Thr^58^/Ser^62^) known to promote c-MYC transcriptional activity.^24^ Gene expression and western blot analysis suggested that this increase in phosphorylation may be because of a higher expression of mRNA and protein levels (Figure 3c and Supplementary Figure S1d). In addition, pathway enrichment analysis against the NCI pathway database revealed that proteins listed within the process ‘validated targets of c-MYC transcriptional activation' were increased in resistant relative to parental cells (Figure 3d and Supplementary Dataset S1).

c-MYC activity contributes to acquired resistance to PI3K inhibitors in other models.^8, 9, 10^ We inhibited c-MYC using two different competitive inhibitors of the c-MYC/MAX interaction (10058-F4; c-MYC inhibitor 1 ^(ref.^^25)^ and 10074-G5; c-MYC inhibitor 2 ^(ref.^^26)^). We found that although in isolation PI3K and c-MYC inhibitors had a modest effect on reducing cell proliferation of resistant cells, concomitant inhibition of PI3K and c-MYC activity reduced cell number by approximately threefold relative to untreated cells (Figure 3e). Surprisingly, inhibition of c-MYC did not prevent the ability of resistant cells to acidify the media (Figure 3f and Supplementary Figure S3c). Overall, our results indicate that although the activities of CAMKII, MEK and c-MYC help resistant cells to survive in a background of chronic PI3K/mTOR inhibition, these proteins do not regulate the metabolic phenotype in these models.^27^

### HIF activity regulates the glycolytic phenotype in PI3Ki-resistant cells

**Figure 4:**
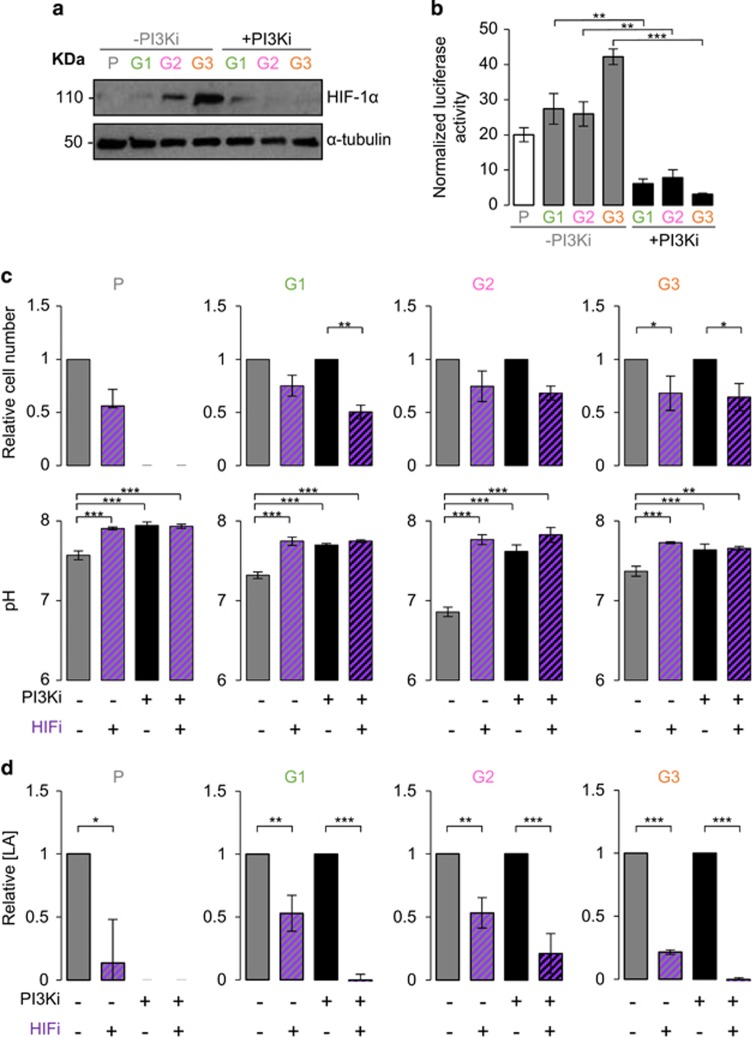
HIF activity is responsible for lactic acid production in PI3Ki-resistant cells during drug holidays. (**a**) HIF-1α protein expression. (**b**) HIF1 transcriptional activity. Data are represented as mean±s.d. (*n*=3, three independent replicates). (**c**) Relative cell numbers and pH values from cell media cultured for 5 days with chetomin (HIFi), PI3Ki or the combination. (**d**) Media lactic acid concentration as a function of treatment for 5 days. For (**c**) and (**d**), data are represented as mean±s.d. (*n*=3, three independent biological replicates). *P*-values calculated using an unpaired, two-tailed Student's *t*-test compared as indicated; *P<0.05; **P<0.01; ***P<0.001.

In addition to c-MYC, bioenergetic metabolic pathways are regulated by the transcription factor HIF.^28^ We found that the process ‘HIF-1α transcription factor network' was also enriched in resistant cells (Figure 3d). We therefore investigated HIF-1α protein expression and found that resistant cells growing in the absence of drug had greater HIF-1α protein than parental cells; this expression decreased dramatically in cells treated with PI3Ki (Figure 4a and Supplementary Figure S4a). Consistent with the immunoblot data (Figure 4a), HIF transcriptional activities were between 3.3- and 13.6-fold greater in resistant cells grown in the absence of the PI3Ki than in its presence (Figure 4b).

To assess the role of HIF in the development^29^ of the hypermetabolic phenotype, we inhibited their activities using chetomin, a disruptor of the HIF/p300 interaction.^30^ Chetomin reduced media acidification to similar levels as when cells were treated with PI3Ki (Figure 4c and Supplementary Figure S4b), and also reduced cell division rates of parental cells; however, G1 and G2 proliferation rates were not significantly affected by inhibition of HIF isoforms (Figure 4c). We observed that HIF inhibition caused a significant decrease in lactic acid content in the media of parental and resistant cells (Figure 4d). Thus, an increase in HIF activities (involving at least HIF-1α), known to act downstream of different oncogenes,^31^ regulates the phenotype of our models of PI3Ki-resistant cells.

### ROS overproduction on drug removal activates HIF and causes a defect on the proliferation of resistant cells that can be recovered by antioxidants

**Figure 5:**
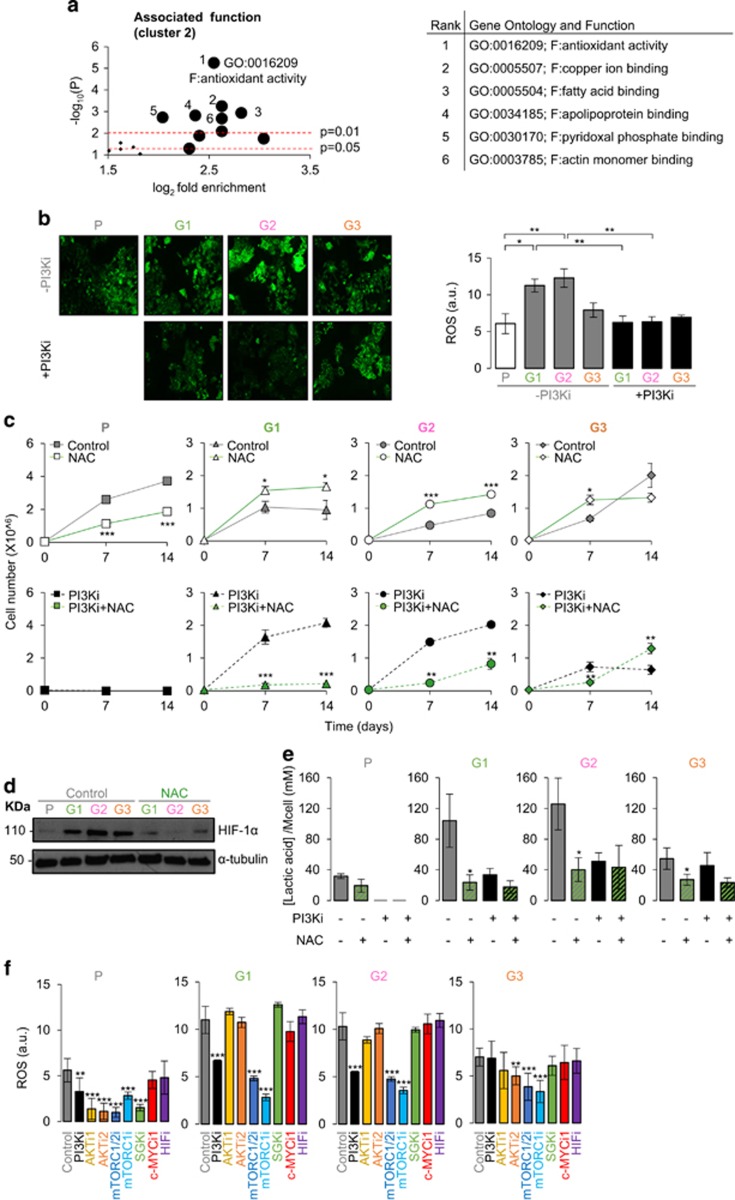
ROS produced during drug holidays in resistant cells activate HIF and cause a proliferative defect that can be recovered by free radical scavengers. (**a**) Gene Ontology analysis of proteins increased in PI3Ki-resistant cells relative to parental (cluster 2 in Figure 3a). (**b**) Representative images and quantification of ROS in cells cultured for 7 days. (**c**) Proliferation of cells as a function of treatment with the named compounds. Media were replaced daily. (**d**) HIF-1α protein expression in cells treated with 200 μM NAC for 5 days. (**e**) Lactic acid produced by cells cultured for 5 days in the absence or presence of vehicle, PI3Ki, NAC or both. Lactic acid form the media were normalized to cell number. (**f**) ROS levels in cells cultured for 7 days with vehicle or inhibitors of the named proteins. Data are mean±s.d. of seven fields per condition. For (**b**), (**c**) and (**e**), data are mean±s.d. of three independent biological replicates. *P*-values calculated using an unpaired, two-tailed Student's *t*-test comparing as indicated for (**b**), and presence or absence of NAC for (**c**) and (**e**). For (**f**), presence or absence of the indicated inhibitor was compared. *P<0.05; **P<0.01; ***P<0.001.

A gene ontology analysis of our phosphoproteomics data showed that antioxidant activity (GO: 0016209) was significantly enriched in resistant cells during drug holidays (Figure 5a). Antioxidant activity may be increased in these cells to counteract by-products of metabolism such as ROS that are expected to increase as a result of high metabolic activity.^32^ To further investigate the involvement of mitochondria in our model, we measured ROS, a natural end product of mitochondrial activity. Untreated G2 and G1 cells produced approximately twofold more ROS than the same cells treated with PI3Ki and parental cells (Figure 5b and Supplementary Figure S5a). PI3Ki-resistant cells increased ROS production during drug holidays but, as noted above (Figure 1), these differed in their extent of metabolic dysregulation.

Because a high intracellular ROS concentration can be toxic,^32, 33^ we hypothesized that increased ROS levels contributed to the defective proliferation of resistant cells. We tested whether reducing ROS with the antioxidant *N*-acetylcysteine (NAC)^34, 35, 36^ would increase the proliferation of resistant cells. After confirming that NAC was a free radical quencher in our model (Supplementary Figure S5b), we found that reduction of ROS by NAC restored the proliferation of cells with elevated metabolism and high ROS levels (G1 and G2 cell lines growing in the absence of PI3Ki;Figure 5c and Supplementary Figure S5b). Conversely, proliferation of cells with lower metabolism and ROS was impaired by NAC (Figure 5c).

Similarly, the lipophilic ROS scavenger α-tocopherol^37^ increased the proliferation of G2 cells growing without PI3Ki, but decreased cell numbers when cultured with PI3Ki (Supplementary Figure S5c). This antioxidant also decreased proliferation of parental cells (Supplementary Figure S5c). Thus, the loss of ROS homeostasis that occurred in G1 and G2 cells after drug removal (probably because of an increase of the OCR; Figure 1f) was responsible for their proliferative defect. In contrast, ROS levels were lower in G3 cells (probably because they did not suffer a significant increase of OCR; Figure 1f) and, consequently, these cells did not experience a proliferative defect. ROS can stabilize HIF.^38^ ROS scavenging with NAC decreased HIF-1α protein expression in the resistant cell lines grown without PI3Ki to that of expression in parental cells (Figure 5d) and significantly reduced the levels of lactic acid in all PI3Ki-resistant cells; this effect being greater in G1 and G2 grown without PI3Ki (Figure 5e).

To delineate in more detail the pathway that leads to ROS production in PI3Ki-resistant cells, we measured cellular ROS after the inhibition of several PI3K pathway members. Treatment of resistant cells that present high oxidative stress (G1 and G2) with PI3Ki, mTORC1/2i and mTORC1i reduced ROS levels, whereas inhibitors of AKT, SGK, CAMKII or MEK were unable to prevent the increase of ROS (Figure 5f and Supplementary Figure S5d). Similarly, neither c-MYC nor HIF inhibition reduced ROS accumulation (Figure 5f and Supplementary Figure S5d).

Together, the data in Figure 5 reinforce the notion that PI3Ki-resistant cells develop an oxidative phenotype during drug holidays that is dependent on PI3K and mTORC1, but does not require AKT, SGK, CAMK, MEK or c-MYC to generate ROS that controls HIF-1α to regulate the glycolytic phenotype.

### The high metabolic activity of resistant cells may be exploited to potentiate the proliferative defect induced during drug holidays

**Figure 6:**
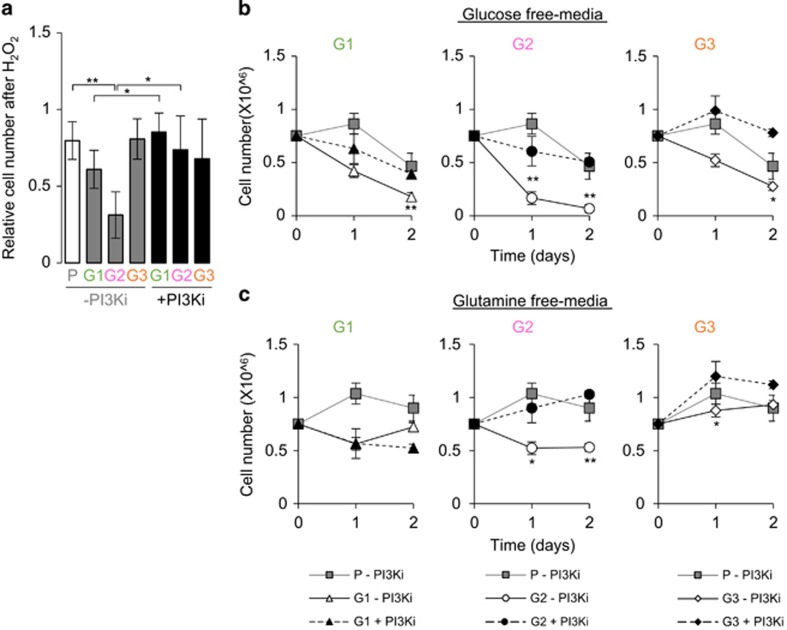
Metabolism as a therapeutic target for PI3Ki-resistant cells during drug holidays. (**a**) Relative cell numbers after 5-day treatment with 130 μM H_2_O_2_ either in the presence or absence of PI3Ki. (**b**) Cell numbers as a function of culture in glucose-free media for 1 and 2 days. (**c**) Cell numbers as a function of culture in glutamine-free media for 1 and 2 days. Data are mean±s.d. of three independent biological replicates. *P*-values calculated using an unpaired, two-tailed Student's *t*-test comparing for (**a**) and (**b**) as indicated, and for (**c**) presence or absence of PI3Ki at days 1 and 2; *P<0.05; **P<0.01; ***P<0.001.

We hypothesized that PI3Ki-resistant cells in drug holidays would be more sensitive to an additional oxidative challenge.^39, 40^ Thus, we used H_2_O_2_ to increase the oxidative stress in sensitive and resistant cells.^41^ Interestingly, H_2_O_2_ significantly reduced the numbers of G1 and G2 cells in growing without PI3Ki to a greater extent than the same cells growing with PI3Ki (Figure 6a).

We then examined whether the elevated metabolic rate was functionally associated with higher nutrient demand. For this purpose, we measured the viability of cells cultured in glucose- and glutamine-free media, as these nutrients serve as primary sources of carbon for adenosine triphosphate production.^42^ The absence of PI3Ki made resistant cells more sensitive to glucose and glutamine withdrawal than those cultured with PI3Ki, and the extent of the sensitivity was proportional to the intensity of the glycolytic phenotype (Figures 6b and c). Resistant cells were also more sensitive to glucose deprivation than parental cells (Figure 6b). Thereby, the hypermetabolic phenotype shown by PI3Ki-resistant cells during drug holidays leads to an increased dependency on nutrients and the higher ROS levels made cells more susceptible to pro-oxidant treatment (Figure 6).

### The extent of metabolic imbalance determines the capacity of PI3Ki-resistant cells to proliferate

**Figure 7:**
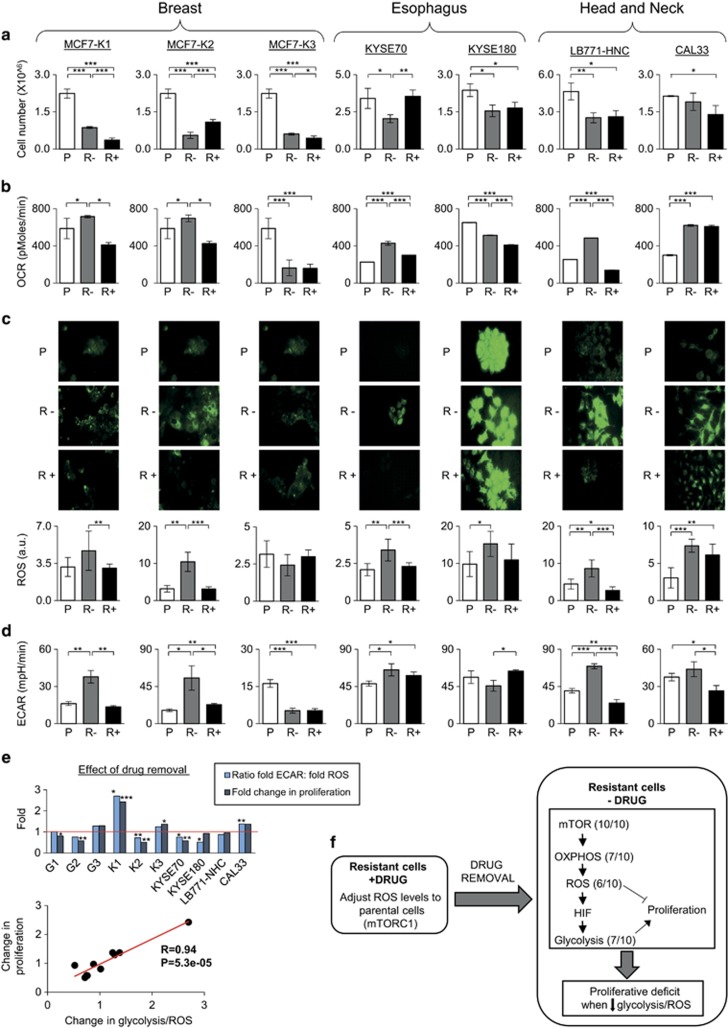
The balance between glycolytic and oxidative metabolic activities determines the proliferation of drug-resistant cells. (**a**) Cell numbers of seven cell lines resistant to different PI3K/mTORC1/2 inhibitors. Cells were cultured in the presence (R+) or absence (R−) of the selection drug. P, parental cells. Data are mean±s.d. of three independent biological replicates. *P*-values were calculated using an unpaired, two-tailed Student's *t*-test comparing conditions as indicated; *P<0.05; **P<0.01; ***P<0.001. (**b**) OCR measured in the same cells as in (**a**). (**c**) ROS measured in the same cells as in (**a**), and (**d**) ECAR measured in the same cells as in (**a**). (**e**) The glycolysis to ROS ratio associates with the proliferation capacity of cells during drug holidays. Change in proliferation was calculated by dividing the number of viable cells remaining after culture in drug holidays by those in cells treated with the specific inhibitor. Ratio of changes in ECAR and ROS was calculated by dividing these values in cells in drug holiday conditions by those of cells growing in the presence of the inhibitors. Values above 1 denote that drug removal had a positive effect on proliferation or glycolysis/ROS ratio (upper panel). The association was statistically significant (bottom panel). *R*, Pearson's correlation value. *P*, *P*-value for Pearson's correlation value. (**f**) Proposed mechanism to explain the proliferative and metabolic alterations that occur in cells with acquired resistance to PI3K/mTOR inhibition during drug holidays.

To elucidate whether bioenergetic remodeling occurs in other models of acquired resistance to PI3K/mTOR inhibition, we analyzed an additional panel of cells, including seven sensitive and resistant cell line pairs derived from breast (MCF7-K1, MCF7-K2 and MCF7-K3), esophagus (KYSE70 and KYSE180) and head and neck (LB771-HNC and CAL33) (Figure 7). These cells were resistant to PI3K/mTOR inhibition and reactivated this signaling pathway following drug removal.^12, 14^ Six out of seven of these resistant cell lines did not recover the growth rate of parental cells after drug withdrawal (Figure 7a). Two of these resistant cell lines (K2 and KYSE70) also proliferated less efficiently when growing without inhibitor in the media than when the compound was present (Figure 7a). Drug holidays resulted in an increase in respiratory activity in five out of seven of the cell lines tested (Figure 7b), and ROS (Figure 7c) and glycolytic activity (Figure 7d) also increased in four of them.

In the panel of 10 resistant cell lines, drug withdrawal led to a reduced proliferation in four of them (G1, G2, K2 and KYSE70) and increase in proliferation in two of them (K1 and K3) (Figure 7e, upper panel). Notably, we found a positive correlation (*R*=0.94, *P*=5.3 × 10^−05^) between cell proliferation and the ratio of glycolytic activity to ROS concentration (Figure 7e, lower panel), suggesting that the toxic effect of ROS accumulation was counteracted by the pro-proliferative contribution of high glycolysis. In summary, Figure 7 indicates that drug removal causes the loss of ROS homeostasis and the increase of the glycolytic pathway in different models of PI3K/mTOR axis independence. The extent of glycolysis and ROS dysregulation and their balance determine the capacity of PI3K/mTOR-resistant cells to proliferate during drug holidays (Figure 7f).

## Discussion

In this study we investigated the biochemical adaptations that allow cancer cells to compensate for chronic PI3K inhibition and how these change during drug holidays. The results obtained from the study of three independent cell lines derived from MCF7 breast cancer cells^14^ were then confirmed in an independent panel of sensitive/resistant models.^12, 14^ In the presence of PI3K pathway inhibitors, resistant cells proliferated at a lower rate than the parental cells from which they originated. This proliferative defect could not be reversed by reactivation of the PI3K/mTOR pathway (Figures 1 and 2). Indeed, some of the resistant cells proliferated less efficiently with an active PI3K pathway (during drug holidays) than in the context of an inactive PI3K pathway (that is, when the PI3Ki was present in the media; Figures 1 and 7). Drug holidays reduced melanoma cells resistant to the BRAF inhibitor vemurafenib but the underlying biochemical causes have remained elusive.^43^ In our models of PI3Ki resistance, drug withdrawal caused a proliferative defect that, interestingly, was associated with the appearance of a metabolic phenotype characterized by increased respiratory and glycolytic activities (Figure 1).

Untargeted proteomics and phosphoproteomics analyses showed that c-MYC, CAMKII and MEK activities were increased in resistant cells. Cell proliferation was inhibited by interfering with c-MYC, CAMKII or MEK activities, suggesting that these proteins contributed to the PI3Ki resistance phenotype. The oncogene c-Myc and the MAPK pathway have been previously implicated in the ability of cells to survive without PI3K.^9, 10, 11^ However, c-MYC, CAMKII or MEK were not involved in the metabolic phenotype of our models. In contrast, this hypermetabolism could be reversed by inhibiting mTORC1/2 or PI3K but not by AKT or SGK inhibitors, implying that, contrary to the canonical understanding of PI3K signaling, these cells possess a PI3K/mTORC1 axis that is independent of AKT. This notion was also supported by phosphoproteomics data showing that PI3K and mTORC1/2 blockade reduced the phosphorylation of a large number of sites that were insensitive to AKT inactivation (Figure 2).

During drug holidays PI3Ki-resistant cells reactivated the PI3K/mTORC1 pathway, which is a positive regulator of mitochondrial respiration,^44, 45, 46^ leading to a dysregulation of ROS homeostasis that in turn hampered resistant cell proliferation (Figure 7f). Resistant cells growing in the presence of drug adapted their ROS levels to those of parental cells (Figure 5), suggesting that a minimum concentration of ROS is required for their proliferation. Free radical accumulation is toxic, whereas at lower concentrations these molecules promote cell proliferation.^47, 48^ However, ROS also act as second messengers to regulate the stabilization of the transcription factor HIF-1α^38^ that induces the expression of metabolic enzymes, leading to an increase in glycolytic activity. Thus, our data imply that altered cellular metabolism is not just a hallmark of oncogenesis, but also occurs during the acquisition of resistance to targeted drug therapy in order to maintain ROS homeostasis.

We observed this high metabolic phenotype in two of the initial set of PI3Ki-resistant cell lines analyzed. To address the question of how frequently this phenotype occurs in cells resistant to PI3K/mTOR inhibition, we investigated whether this mechanism also applied to three breast cancer cell lines resistant to Ku-0063794 and to four models of resistance to BYL719 (two of which were derived from esophagus and two from head and neck cancers). After drug removal, 9/10 of these models did not recover the proliferative capacity of parental cells despite reactivating mTORC1. Respiration, ROS production and glycolytic activity were increased in seven, six and seven of these models, respectively. Despite the variability in the extent of the metabolic dysregulation, a common feature across all the resistant models analyzed was that these showed a remarkable association between cell proliferation and the ratio of glycolytic activity to ROS levels (Figure 7e). This observation supports the notion that the metabolic status of PI3Ki-resistant cells determines their ability to proliferate.

The hypermetabolic phenotype suggests new therapeutic opportunities as adjuvant to the drug holiday strategy. Increased glycolysis during drug holidays rendered resistant cells more sensitive to nutrient starvation, and high levels of free radicals increased their sensitivity to further oxidative stress (Figure 6). Drugs that cause an intracellular accumulation of lactic acid levels, such as inhibitors of the monocarboxylate transporters^49^ or compounds that inhibit free radical scavengers,^32, 50^ could increase the therapeutic window of these compounds, enhancing the ability of the drug holiday strategy to delay the occurrence of resistance to PI3Ki.

## Materials and methods

Details of reagents used in this study, including compounds, media, antibodies and nutrients are given in Supplementary Tables S1–S4.

### Cell lines

MCF7 breast cancer cell line was obtained from ATCC (HTB-22; Manassas, VA, USA). Three cell lines resistant to GDC-0941 (G1-G3) or to Ku-0063794 (K1-K3) were obtained by exposing MCF7 cells to increasing concentrations of the respective compound during 6 months.^14^ Cell pairs sensitive or resistant to BYL719, namely CAL-33, LB771-HNC, KYSE180 and KYSE70, were a kind gift from Dr Baselga (Human Oncology and Pathogenesis Program, Memorial Sloan Kettering Cancer Center, New York, NY, USA).^12^ Cells were maintained in humidified incubator with 5% CO_2_ using culture media Dulbecco's modified Eagle's medium supplemented with 10% fetal bovine serum, 100 U/ml penicillin and 1 μM of the respective compound, except when indicated (see Supplementary Tables S1 for details of media and compounds used for cell culture).

### Cell proliferation assay

The number of viable cells was assayed using a Vi-CELL cell counter (Beckman Coulter, Inc., Brea, CA, USA). A total of 2000 cells on 96-well plates were analyzed by addition and 30 min of incubation of the Guava Nexin reagent (EMD Millipore, Billerica, MA, USA) or the Guava Cell Cycle reagent (EMD Millipore). Then, 2000 events (apoptosis assays) or 5000 events (cell cycle distribution) were acquired per sample and FSC files were analyzed using CytoSoft software (Guava Technologies, Billerica, MA, USA).

### Metabolic assays

Oxidative capacity was measured via the MTS assay after 2 h of incubation (Promega, Madison, WI, USA). To measure basal OCR and ECAR, 80 000 cells per well were incubated for 24 h at 37 °C in 5% CO_2_ with XF assay media (Agilent technologies, Santa Clara, CA, USA). To establish OCR and ECAR values, four readings were acquired per experiment (Seahorse Bioscience, Santa Clara, CA, USA). Culture media pH was measured using a pH meter (Hanna Instrument). For lactate measurements, lactate was extracted from the media after 5 days of culture and subsequently analyzed by TSQ Vantage mass spectrometer (Thermo Fisher Scientific, Waltham, MA, USA). The parental ion mass followed on a negative mode was 89.0244 Da. For quantification extracted ions chromatograms for the parental ion were obtained and the area under the curve was measured using Excalibur software (version 2, Thermo Fisher Scientific).

### ROS measurement

ROS were assayed by addition of 10 μM dichloro-dihydro-fluorescein diacetate (DCFH-DA, Sigma-Aldrich, St Louis, MO, USA) for 30 min, and they were further washed with cold phosphate-buffered saline and observed under a Nikon eclipse CiS/Ci-L microscope and process with Nis elements D imaging software (Nikon Instruments, Amsterdam, The Netherlands). Quantification of ROS was done with Image J 1.48V (National Institutes of Health, Bethesda, Maryland, USA). The mean intensity of pixel was calculated for each image. Data for each biological replicate are the mean of seven fields per condition.

### HIF transcriptional activity

Cells at 70% confluence were transfected with of a plasmid (30 ng per well). At 2 days after transfection, cells were lysed for 20 min using 1 × passive lysis buffer (Promega). Reporter activation was determined using the Nano-Glo Dual-Luciferase Reporter Assay System (Promega). Luciferase activity was measured using a Dynex Revelation 4.06 luminometer (Dynex Technologies, Chantilly, VA, USA).

### Microfluid expression of metabolic genes

Expression of 96 metabolic genes was quantified on the multiplex Fluidigm platform following the manufactures' instructions.

### Mass spectrometry-based proteomics and phosphoprotemics

Cells from four independent biological replicates were lysed using urea lysis buffer containing phosphatase inhibitors (1 mM Na_3_VO_4_, 1 mM NaF, 1 mM β-glycerol-phosphate and 2.5 mM Na_4_P_2_O_7_). Extracted proteins were digested with trypsin and peptides analyzed in two technical replicates on an LTQ-Orbitrap mass spectrometer (Thermo Fisher Scientific). For phosphoproteomics experiments, TiO_2_ enrichment method was used in two independent biological replicates and run in two technical replicates in an LTQ Orbitrap or in a Q Exactive Plus mass spectrometer (Thermo Fisher Scientific). Mascot was used to identify peptides from tandem mass spectrometry data. Pescal was used for label-free quantification.^14^ Normalized peak areas of peptides or phosphopeptides were averaged between replicates and fold change between conditions was calculated. Kinase activity was estimated from phosphoproteomics data using the kinase substrate enrichment analysis approach as previously described^4^ (see Supplementary Materials and methods). Data are available via ProteomeXchange with identifier PXD003594.

### Statistical analysis

All data represent the mean±s.d. of a minimum of three independent experiment. The resulting data were considered significant when the *P*-value was <0.05. Significance of enrichment of kinase substrates, ontologies and pathways in each cluster was determined by the hypergeometric test. For cell viability assays, bioenergetic assays, microscopic fluorescence data and proteomics data, paired Student's *t*-tests were performed following the assumption of normal distribution and equal variance of the data.

### Western blot

After washing with phosphate-buffered saline containing 1 mM Na_3_VO_4_ and 1 mM NaF, cells were lysed with a Triton base buffer supplemented with protease inhibitors (Complete Mini EDTA-free; Roche, Basel, Switzerland) and phosphatase inhibitors (1 mM Na_3_VO_4_, 1 mM NaF, 1 mM β-glycerol-phosphate, 2.5 mM Na_4_P_2_O_7_ and 1 μM okadaic acid). Cell extracts were maintained during 30 min on ice. Proteins were quantified by BCA (Thermo Fisher Scientific) and separated by gradient gels. Primary antibodies (see Supplementary Table S3 for details) were incubated overnight at 4 °C and secondary antibodies were incubated for 1 h. Detection was by enhanced chemiluminescence (Thermo Fisher Scientific).

## Supplementary Material

Supplementary Informations
