# Supplementary material for: Oxidative stress downstream of mTORC1 but not AKT causes a proliferative defect in cancer cells resistant to PI3K inhibition
Source: Oncogene. 2016 Dec 19;36(19):2762–74. doi: 10.1038/onc.2016.435 (PMC5362070; doi:10.1038/onc.2016.435)
Supplement: Supplementary Informations [file onc2016435x1.docx]

**SUPPLEMENTARY INFORMATION**

**Oxidative stress downstream of mTORC1 but not AKT causes a proliferative defect in cancer cells resistant to PI3K inhibition**

Maria Dermit, Pedro Casado, Vinothini Rajeeve, Edmund H. Wilkes, Daniel E. Foxler, Hayley Campbell, Susan Critchlow, Tyson V. Sharp, John G. Gribben, Robert Unwin, Pedro R. Cutillas.

**SUPPLEMENTARY TABLES**

**Supplemental Table S1.** Details and concentrations of compounds used.

**Supplemental Table S2.** Details and concentrations of media used.

**Supplemental Table S3.** Details and concentrations of antibodies used.

**Supplemental Table S4.** Details and of nutrients used.

**SUPPLEMENTARY DATASET**

**Supplemental Dataset S1.** Details of proteins significantly modulated indentified in this study.

**Supplemental Dataset S2.** Details of phosphoproteins significantly modulated indentified in this study.

**SUPPLEMENTARY FIGURES**

**Supplemental Fig.1.** Description of cellular and metabolic features of MCF7 cells resistant to PI3K inhibition

**Supplemental Fig. 2.** Signaling adaptations in MCF7 cells resistant to PI3K inhibition.

**Supplemental Fig. 3.** Role of CAMKII, MEK and c-MYC signaling pathways on the resistance to PI3K inhibition and the development of the metabolic phenotype.

**Supplemental Fig. 4.** Role of HIF on the metabolic phenotype developed by PI3K inhibitor resistant cells.

**Supplemental Fig. 5.** Modulation of ROS concentration in response to different compound treatments and effect of ROS levels in cell proliferation

**SUPPLEMENTARY MATERIAL AND METHODS**

**SUPPLEMENTARY TABLES**

**Supplemental Table S1.** Details and concentrations of compounds used

| **Name** | **Brand** | **Reference** | **Dilution** | **Concentration used** | **Primary target** |
| --- | --- | --- | --- | --- | --- |
| GDC-0941 | Chemdea | CD0245 | DMSO | 1 μM | PI3K α/δ |
| Ku-0063794 | Chemdea | CD0274 | DMSO | 1 μM | mTORC1/2 |
| MK-2206 | Selleckchem | S1078 | DMSO | 1 μM | AKT 1/2/3 |
| A-VII | Cayman | 14870 | DMSO | 1 μM | AKT 1/2 |
| Everolimus | Selleckchem | S1120 | DMSO | 20 nM | mTORC1 |
| 10058-F4 | Sigma | F3680 | DMSO | 25 μM | c-MYC |
| 10074-G5 | Sigma | G3798 | DMSO | 20 μM | c-MYC |
| GSK-650394 | Sigma | 650394 | DMSO | 0.5 μM | SGK |
| Trametinib | Selleckchem | S2673 | DMSO | 0-20 μM | MEK |
| KN-93 | Tocris | 139298 | DMSO | 0-200 μM | CAMKII α/β/γ/δ |
| N-acetylcysteine | Sigma | A0737 | Water | 200 μM | Free  radicals |
| (±)-α-Tocopherol | Sigma | T3251 | DMSO | 0-20 μM | Free  radicals |
| H_2_O_2_ | Sigma | 216763 | Water | 130 μM | Free  radicals |
| BYL719 | Selleckchem | 2814 | DMSO | 1 μM | PI3Kα |

**Supplemental Table S2.** Details and concentrations of media used.

| **Name** | **Brand** | **Reference** | **Glucose [mM]** | **Glutamine [mM]** | **Sodium pyruvate**  **[mM]** |
| --- | --- | --- | --- | --- | --- |
| DMEM | Sigma | 6429 | 25 | 4 | 1 |
| DMEM   no glucose, no phenol red, no glutamine | Life  technologies | A14430 | 0 | 0 | 1 |
| XF assay medium | Seahorse Biosciences | 102365-100 | 25 | 2 | 1 |
| RPMI 1640 | Sigma | 8750 | 11.10 | 2 | 0 |

**Supplemental Table S3.** Details and concentrations of antibodies used.

| **Name** | **Brand** | **Reference** | **Concentration** | **Procedure** |
| --- | --- | --- | --- | --- |
| FASN | Cell Signalling Technologies | 3189 | 1/1000 | Western blot |
| LDHB | Abcam | ab53292 | 1/1000 | Western blot |
| p-PRAS40 Ser^246^ | Cell Signalling Technologies | 2640 | 1/2000 | Western blot |
| p-4EBP1 Thr^37^/Thr^46^ | Cell Signalling Technologies | 2855 | 1/1000 | Western blot |
| p-AKT Ser^473^ | Cell Signalling Technologies | 4058 | 1/1000 | Western blot |
| c-MYC | Cell Signalling Technologies | 5605 | 1/1000 | Western blot |
| HIF-1α | Cell Signalling Technologies | 3716 | 1/500 | Western blot |
| α-Tubulin | Cell Signalling Technologies | 2144 | 1/1000 | Western blot |

**Supplemental Table S4.** Details and of nutrients used.

| **Name** | **Brand** | **Reference** |
| --- | --- | --- |
| D-(+)-Glucose | Sigma | G7528 |
| L-Glutamine | Sigma | G7513 |
| Sodium pyruvate solution | Sigma | S8636 |

**Supplemental Dataset S1.** Details of proteins significantly modulated indentified in this study. (A) Log 2 fold changes of proteins that are modified in resistant cells in comparison to parental and proteins modified in resistant cells in response to removal of the selection drug (GDC-0941 1 μM). (B) Significance of the modifications presented in (A).

**Supplemental Dataset S2.** Details of phosphoproteins significantly modulated indentified in this study. (A) Log 2 fold changes of phosphopeptides that are modified in resistant cells in comparison to parental and phosphopeptides modified in resistant cells in response to removal of the selection drug (GDC-0941 1 μM). (B) Significance of the modifications presented in (A).

**SUPPLEMENTARY FIGURES**

**Supplemental Figure 1. Description of cellular and metabolic features of MCF7 cells resistant to PI3K inhibition**. (**a**) Cell numbers as a function of proliferation for parental and resistant cells measured using the crystal violet assay. Data are represented as mean ± SD (*n*=3, three independent technical replicates). P-values were calculated using an unpaired, two-tail Student’s t-test against control (DMSO); * *P* < 0.05; ** *P* < 0.01; *** *P* < 0.001. (**b**) Cell numbers and pH values for parental and resistant cells cultured for 10 days in the presence or absence of GDC-0941 (+PI3Ki) or for 5 days with vehicle control (-PI3Ki) followed by 5 days with GDC-0941 (+PI3Ki ->-PI3Ki). (**c**) heatmap representing the expression values for 96 metabolic genes quantified by Fluidigm platform. The dendrogram shows the hierarchical clustering of differentially expressed genes. Graphs show averaged normalized expression for 6 representative genes. (**d**) Cell cycle distribution for parental and resistant cells. Data are mean ± SD (*n*=3, three independent replicates). (**e**) Percentage of apoptosis for parental and resistant cells after culture for 5 days in the absence or presence of 1 µM GDC-0941. Data are represented as mean ± SD (*n*=3, three independent technical replicates). P-values were calculated through an unpaired, two-tail Student’s t-test comparing presence and absence of GDC-0941; * *P* < 0.05; ** *P* < 0.01; *** *P* < 0.001. F, protein content for parental and resistant cells maintained in culture for 5 days in the absence or presence of PI3Ki. Data are represented as mean ± SD (*n*=3, three independent biological replicates). P-values were calculated using an unpaired, two-tail Student’s t-test comparing presence and absence of GDC-0941; * *P* < 0.05; ** *P* < 0.01; *** *P* < 0.001.

**Supplemental Figure 2. Signaling adaptations in MCF7 cells resistant to PI3K inhibition.** (**a**) Replicated western blots of those shown in Fig 2A assessing reactivation of the PI3K/AKT/mTORC axis upon drug withdrawal. (**b**) Scheme for the phosphoproteomics experiment shown in Fig 2D. (**c**) extracted ion chromatograms (XICs) for the peptides p-EIF4E-BP2 (Ser^44^/Thr^45^) at m/z 1073.4843 and p-PRAS40 (Thr^246^) at m/z 516.7240. XICs were created from MS data obtained for the experiment detailed in Fig 2B. Shown are the temporal elution profiles of the first (blue line), second (red line), and third (green line) isotopes. Vertical dotted lines represent the actual or predicted retention time for the phosphopeptide in each sample. Control; GDC-0941, PI3K inhibitor; MK-2206, AKT inhibitor; Ku-0063794, mTORC1/2.

**Supplemental Figure 3.** **Role of CAMKII, MEK and c-MYC signaling pathways on the resistance to PI3K inhibition and the development of the metabolic phenotype**. (**a**) Scheme used to analyze the phosphoproteomes of cells grown in the presence or absence of GDC-0941. (**b**) Inferred MEK and CAMKII activities using KSEA. (**c**) Media images of cells grown in the presence or absence of c-MYCi1 (10058-F4), PI3Ki (GDC-0941), CAMKII (KN-93) or MEKi (trametinib) as indicated.

**Supplemental Figure 4.** **Role of HIF on the metabolic phenotype developed by PI3K inhibitor resistant cells**. (**a**) optical densities (OD) of the HIF-1α band in the Western blot shown in Figure 4A. ODs for the HIF-1α bands were normalized to those of tubulin. (**b**) media images of cells grown in the presence or absence of HIFi (chetomin) or PI3Ki (GDC-0941) as indicated.

**Supplemental Figure 5. Modulation of ROS concentration in response to different compound treatments and effect of ROS levels in cell proliferation.** (**a**) Images of ROS as determined by fluorescence emission of DCFH-DA. Cells were grown in the presence or absence of GDC-0941 for 7 days. (**b**) Schematic representation of experiments assessing the effect of NAC administration on parental and resistant cells (left image). Cell numbers and quantification of ROS after 7 day treatment with NAC (right image). Cell number data are represented as mean ± SD (*n*=3, three independent biological replicates). ROS values are mean ± SD of three images. P-values were calculated using an unpaired, two-tail Student’s t-test comparing as indicated. (**c**) Relative cell numbers as a function of treatment with α-tocopherol. Parental cells were exposed to increasing concentrations of α-tocopherol (grey line) and resistant cells were exposed to increasing concentrations of α-tocopherol in the presence (dash line) or absence (continuous line) of GDC-0941. Before α-tocopherol treatment, resistant cells were grown for 7 days in the presence or absence of GDC-0941. Cell proliferation was assessed using the crystal violet assay. Data are represented as mean ± SD (*n*=3, three technical replicates). P-values were calculated through an unpaired, two-tail Student’s t-test comparing as indicated; * *P* < 0.05; ** *P* < 0.01; *** *P* < 0.001. (**d**) Representative images of ROS staining with DCFH-DA in cells after 7 day treatment with vehicle, AKTi2 (MK-2206), mTORC1 (everolimus), HIFi (chetomin), c-MYCi1 (10058-F4), CAMKII (KN-93) or MEKi (trametinib).

**SUPPLEMENTARY MATERIAL AND METHODS**

**Proteomics Mass Spectrometry**

The proteomics analysis of sensitive and resistant MCF7 cells in basal conditions and phosphoproteomics analysis of the G2 cells in the presence of inhibitors of the PI3K pathway were run in a nano flow ultrahigh pressure liquid chromatography (UPLC, nano Acquity, Waters) coupled to an LTQ-Orbitrap XL mass spectrometer (Thermo Fisher Scientific). The phosphoproteomics study of sensitive and resistant MCF7 cells in basal conditions was run in a Dionex UltiMate 3000 RSLC nano coupled to an Orbitrap Q Exactive Plus mass spectrometer (Thermo Fisher Scientific). For the LTQ-Orbitrap system, proteomics sample pellets were suspended in 200 µL of reconstitution buffer 1 (0.1% TFA) while phosphoproteomics pellets were reconstituted in 12 µL of the same buffer. Then, 4 μL were loaded in the system. For LC separation, solvent A (0.1% FA) and solvent B (100% ACN +0.1% FA) were used as mobile phases. Peptides were loaded in a nano ACQUITY UPLC Symmetry C18 Trap Column (catalog no 186006527) for 8 min with a nano flow of 2 µL/min. Peptides were separated using a ACQUITY UPLC Peptide BEH C18 nano ACQUITY Column (catalog no 186003543). For peptide elution, it was established a gradient from 5 to 35% of solvent B during 150 min with a background pressure of 4,000 psi and a nano flow of 0.3 µL/min. Lastly, the column was washed with 35- 85% B for 10 min and equilibrated with 1% B for 15 min. Peptides were transferred to the online copulated LTQ-Orbitrap XL system. Full scan survey spectra (m/z 375-1,800) were acquired with 30,000 resolution at m/z 400. The 5 most intense ions for each full MS scan were selected in a data-dependent manner for collision induced dissociation (CID) and MS/MS scanning (50-2,000 m/z). The fragmentation was performed at 35% normalized energy collision. In the data-dependent acquisition, a 30 s dynamic exclusion was enabled with an exclusion list of 500 entries and 10 ppm mass window. This produced a duty cycle of 2.5 s. For the Q Exactive Plus system, sample pellets were resuspended in 20 μL of reconstitution buffer 2 (3% ACN; 0.1% TFA). For the LC solvent A (0.1% FA) and solvent B (100% + 0.1% ACN) were used as mobile phases. 4 μL of reconstituted phosphopeptide solution were loaded in a μ-precolumn (catalog no 160454) for 2 min at a back pressure of 15 bar and a nanoflow of 10 µL/min. Then phosphopeptides were separated in an Acclaim PepMap 100 column (catalog no 164569) using a gradient that went from 3% to 23% B during 120 min with a background pressure of 155 bar and a nanoflow of 0.3 µL/min. Finally, the column was washed with 23-85% B for 7 min and equilibrated with 3% B for 3 min. Phosphopetides were transferred to the online connected to a Q Exactive Plus system. Full scan survey spectra (m/z 375-1,500) were acquired with a 70,000 resolution. The 20 most intense ions for each full MS scan were selected using an isolation width of 1.6 Da in a data-dependent manner for higher energy collisional dissociation (HCD) and MS/MS scanning (200-2,000 m/z) with a resolution of 17,500. In the data-dependent acquisition, a 30 s dynamic exclusion was enabled with an exclusion list of 10 ppm mass window. This produced a duty cycle of 2.1 s. For phosphoproteomics and proteomics analysis, chromatographic peaks were about 30 s at the base which allowed the construction of extracted ion chromatograms (XICs) with least 10 data points.

**Lactic acid measurement**

Lactic acid was extracted from 100 µL of media collected after 5 days of cell culture. Samples were mixed and deproteinized with 600 µL methanol: acetonitrile (1:1) and centrifuged at 4°C, during 10 min at 14,000 rpm. The supernatant was dried out in a speedvac overnight and pellets were stored at -20°C until the MS analysis. Running time was set up for 6 min and column eluents analyzed in a TSQ Vantage mass spectrometer (Thermo Fisher Scientific). The parental ion mass followed on a negative mode was 89.0244 Da. For quantification extracted ions chromatograms for the parental ion were obtained and the area under the curve was measured using Excalibur software (version 2, Thermo Fisher Scientific). Area values were extrapolated to a standard curve with range 0-200 mM.

**Proteomics Data Analysis**

Protein identification from the MS/MS data was automated with Mascot Daemon 2.5.0. Mascot Distiller 2.5.1.0 was used to generate peaklist files (MGFs) and Mascot 2.5 search engine was used to match peaks to peptides comprised in proteins annotated in the SwissProt Database (SwissProt_2012Oct.fasta for proteomics or uniprot_sprot_2014_08.fasta for phosphoproteomics analysis) with a FDR of ~1%. For protein searches, two variable modifications for proteomics (PyroGlu on N-terminal Gln; and oxidation of Met) or five variable modifications for phosphoproteomics analysis (previous 2 modifications plus Phosphorylation on Ser, Thr, and Tyr), one fixed modification (carbamidomethyl Cys) and two trypsin miss cleavages were allowed. A mass tolerance of 10 ppm for the MS scans and 25 mmu for the MS/MS scans was permitted. For label-free peptide quantification, Pescal software was used to construct the XICs for all the peptides identified across all samples. The XIC windows were 7 ppm and 2 min. Normalized peak areas from XICs were averaged between replicates and fold change between conditions was calculated. Statistical significance was assessed with a student’s t-Test. The mass spectrometry proteomics data have been deposited to the ProteomeXchange Consortium via the PRIDE partner repository with the dataset identifier PXD003594 and 10.6019/PXD003594.

**KSEA and Clustering Analysis**

KSEA analysis was performed as previously describe (4). Briefly, phosphopeptides were grouped into substrate groups linked to particular kinases obtained from the PhosphoSite database or from in-house experiments. Z-scores for each kinase were calculated as (mS−mP) *m1/2/d, where mS is the log2 of the mean abundances for each kinase group, mP is the log2 of the mean abundances of the whole data set, m is the size of each substrate group and d is the SD of the mean abundances of the entire data set. Excel software was used to transform Z-scores into P-values. Phosphopeptides or proteins (in phosphoproteomics or proteomics experiments, respectively) showing statistically significant differences in at least one condition were classified into groups using k-means clustering. Significance of enrichment of kinase substrates, ontologies and pathways in each cluster was determined by the hypergeometric test the extent of enrichment by the formula e=log2([a/b]/[c/d]), where a is the number of entries that belongs to a given ontology, pathway or group in a given cluster, b is the number of entries in the cluster, c is the number of entries that belongs to the ontology, pathway or group and d is the total number of entries. These algorithms were coded in VBA.
